# Essential Oils from Residual Foliage of Forest Tree and Shrub Species: Yield and Antioxidant Capacity

**DOI:** 10.3390/molecules26113257

**Published:** 2021-05-28

**Authors:** Irene Mediavilla, Eva Guillamón, Alex Ruiz, Luis Saúl Esteban

**Affiliations:** 1 Centre for the Development of Renewable Energies—Centre for Energy, Environmental and Technological Research, CEDER-CIEMAT, Autovía de Navarra A-15, Salida 56, 42290 Lubia, Spain; luis.esteban@ciemat.es; 2 Centre for the Food Quality, INIA, C/Universidad s/n, 42004 Soria, Spain; guillamon.eva@inia.es; 3 CHROMESSENCE, C./Pompeu Fabra, 25, 05530 La Garriga, Spain; alex.ruiz@chromessence.com

**Keywords:** antioxidant activity, *Cistus ladanifer*, essential oil, *Eucalyptus globulus*, forestry biomass foliage

## Abstract

Increasing applications and markets for essential oils could bring new opportunities for cost-effective and sustainable management of unused forestry biomass; however, better knowledge of the production and application of such essential oils is necessary. The objective of this work is to contribute to greater knowledge of the essential oil production on a pilot scale from foliage biomass of wild shrubs and tree residues produced in some forestry enhancement operations and to study their antioxidant capacity (ORAC—oxygen radical absorbance capacity). Fresh biomass (twigs) of seven species (*E. globulus*, *E. nitens*, *P. pinaster*, *P. sylvestris*, *R. officinalis*, *C. ladanifer*, and *J. communis*) was manually collected in Spain in two different periods and was ground at 30 mm and distilled in a 30 L stainless steel still with saturated steam. The essential oil components were identified by GC–MS and quantified by GC–FID, and their antioxidant activity was determined with the ORAC method. Promising results on essential oil yield were obtained with *E. globulus*, *E. nitens*, *R. officinalis,* and *J. communis*. All essential oils studied exhibited antioxidant capacity by the ORAC assay, particularly that from *C. ladanifer.* Moreover, oxygenated sesquiterpenes contents, one of the minor components of oils, were significantly correlated with ORAC values.

## 1. Introduction

According to the EU’s official soil database [1], five Mediterranean countries have over 50% of the EU28′s shrublands, i.e., 16 Mha, of which slightly above half (8.4 Mha) are located in Spain. Currently, this large extent of shrubland is the result of several factors, namely, forest fires, abandonment of low productive cropland and/or extensive grazing, and global warming allowing sclerophyllous shrub species, which best cope with drought and high temperatures, to flourish [2].

Shrubs are colonizing pioneer plants in abandoned agricultural and grazing lands, whose biomass resources are barely or marginally exploited, and where wildfires commonly consume the biomass contained in such lands. Mediterranean shrubs are generally well adapted to fire (pyrophytic species) and their widespread distribution is in fact favored by fire-prone environments [3,4]. Nonetheless, periodic fires result in progressive soil impoverishment, erosion, and loss of organic matter, together with environmental pollution, loss of human life, and vast economic losses as unwanted collateral impacts [4,5].

If climate change forecasts are taken into account, fire-prone conditions are predicted to increase across the world and especially in territories with Mediterranean climates, such as the Mediterranean Basin, the North American West Coast, and Australia. Scientists agree that climate change will drive vegetation shifts toward drought-resistant species [2,6]. In this respect, and especially in Mediterranean regions, adaptation means maintaining forest fuel densities under environmentally acceptable levels that allow us to ensure that the risk and virulence of forest fires are as low as possible [7]. Therefore, forestry management by clearing scrub and tree vegetation must be a priority in fire-fighting plans. However, clearing, pruning, and forest enhancement operations, in general, are costly and require a great amount of economic resources. In that sense, scientists and experts agree that a paradigm shift is needed from the current approach, which is based primarily on extinguishing fires and costly preventive silviculture, toward a new model based on cost-effective forest management [8]. To this end, it is necessary to develop highly added-value bioproducts and biofuels from the biomass obtained in forest enhancement or preventive operations while generating new incentives for rural development and fighting depopulation.

Valuable essential oils can be obtained from species produced in the forest of the Mediterranean area [9]. Essential oils are raw materials for potential cosmetic and pharmaceutical products but could also be used as biocides and preservatives to replace today’s chemical products [10,11], which are more harmful and long lasting.

**Figure 1:**
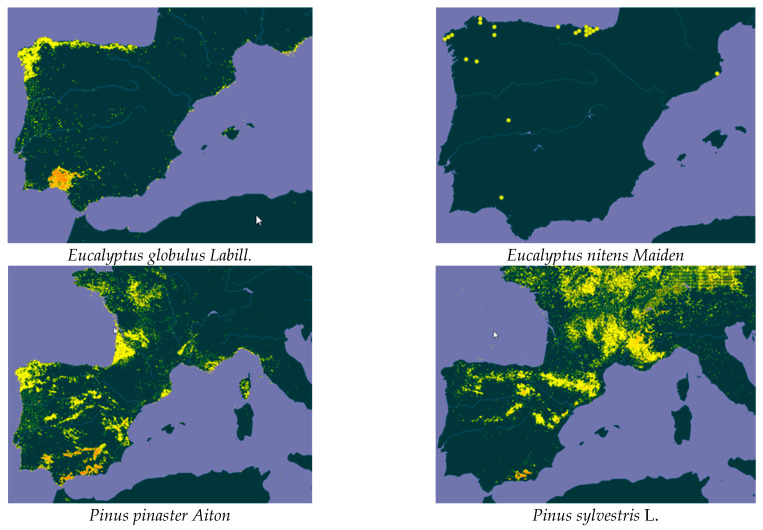

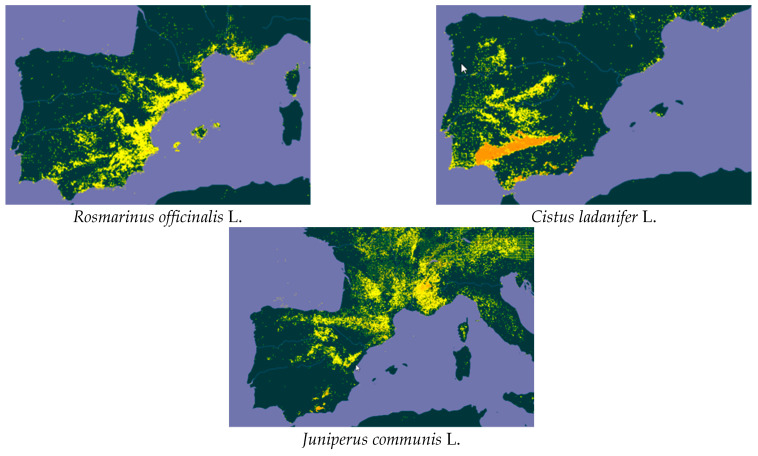
Distribution of the selected species in the Mediterranean area according to the Global Biodiversity Information Facility (GBIF) [12].

The high and increasing market value of essential oils could allow the exploitation of forest resources to be economically viable. Among the species considered of interest from the point of view of their essential oil content and biomass products, seven were selected in this work due to their high availability in both wild and cultivated forms. Four of them were tree species—*Eucalyptus globulus Labill.*, *Eucalyptus nitens Maiden*, *Pinus pinaster Aiton*, and *Pinus sylvestris* L.—and three of them were shrub species—*Rosmarinus officinalis* L., *Cistus ladanifer* L., and *Juniperus communis* L. (Figure 1).

In Spain, according to data from the Spanish Forest Map [13,14], the distribution area of most of the species being studied is considerable. The largest surface area is covered by *C. ladanifer* (1.7 Mha), followed by *P. pinaster* (1.35 Mha), *R. officinalis* (1.3 Mha), *P. sylvestris* (1.25 Mha), *E. globulus* (625,000 ha), *J. communis* (60,000 ha), and finally, *E. nitens*, with 12,000 ha. The surface area of *E. nitens* may be larger since it is a species that is commonly used in new wood production plantations in northern Spain.

Different studies related to the essential oil production from the above-mentioned species have been reported in the literature. Nevertheless, most of them have been carried out on a laboratory scale, making it difficult to extrapolate the published results to an industrial scale.

With regard to the application of the different essential oils studied in this work, rosemary essential oil is the most widely used on the market due to its applications in the food and cosmetics industry [15], followed by rockrose in the cosmetics industry [16], and juniper in the food, pharmaceutical, and cosmetics industries [17]. However, all the essential oils analyzed have proven properties, such as the high antibacterial activity of eucalyptus [18], the high antioxidant power of juniper (both berries and leaves) [19,20], the antimicrobial activity of pine oil [21], and the herbicidal activity of rockrose [22], and therefore, an effort should be made to make this source of natural products available on the market.

Recently, antioxidant activity from natural sources, such as essential oils from plants, has received increasing attention, particularly in the food industry. Dietary antioxidants can stimulate cellular defenses and reduce oxidative damage or stress related to many human diseases such as cancer, atherosclerosis, cardiovascular and neurological diseases, as well as the aging process [23,24,25]. In addition, antioxidant components from essential oil contribute to extending food shelf life [26,27]. Terpenes compounds are the most common constituents of essential oils, and their effectiveness as antioxidants has been widely demonstrated in vivo and in vitro assay [27].

The objective of this research is twofold—on the one hand, to contribute to greater knowledge of the essential oil production on a pilot scale from foliage biomass of wild shrubs and tree residues produced in some forestry enhancement operations, and on the other hand, to study the antioxidant capacity (ORAC—oxygen radical absorbance capacity) of the essential oils obtained.

## 2. Results

### 2.1. Essential Oil Extraction

Fresh biomass collected manually was ground at 30 mm and distilled.

The essential oil yield obtained in the batch distillation tests, expressed in weight percentage and referred to dry plant, is shown in Table 1. Moreover, the *p*-values of the F-tests corresponding to the variance analysis used to determine differences across the collection period within the same species are also included.

Significant (*p* < 0.05) differences were found between the average yields when the two collection periods were considered within the same species, with the exception of *C. ladanifer* and *P. sylvestris*. Comparing the yields obtained for the different species, *E. globulus* is the species with the highest essential oil yield, followed by *E. nitens*, *R. officinalis*, *J. communis*, *P. pinaster*, *P. sylvestris*, and finally, *C. ladanifer*.

### 2.2. Essential Oil Composition

The essential oils corresponding to the same species and collection period were blended and analyzed (see Section 4.3). Table 2 includes the main component groups identified and their quantification considering the relative area percentage.

Monoterpene hydrocarbons were the main component of the essential oils of *C. ladanifer* (62.67 and 49.46%), *J. communis* (59.19 and 79.58%), *P. pinaster* (67.90 and 60.57%), and *P. sylvestris* (74.24 and 84.84%). On the other hand, the essential oils of *E. globulus* and *E. nitens* showed high contents of oxygenated monoterpenes (52.19 and 68.85% for *E. globulus*, and 79.58 and 80.46% for *E. nitens*). Finally, high contents of both monoterpene hydrocarbons and oxygenated monoterpenes were remarkable in the *R. officinalis* essential oil, with values of 46.01 and 37.75% for monoterpene hydrocarbons and 43.41 and 53.95% for oxygenated monoterpenes. With regard to sesquiterpene hydrocarbons, the sample corresponding to the *J. communis* essential oil of the first sampling period showed the highest value (34.20%), followed by the sample corresponding to the *P. pinaster* essential oil of the second sampling period (33.53%). Concerning the oxygenated sesquiterpenes, the highest values were analyzed in the essential oils of *C. ladanifer* (9.47 and 15.35%).

The main components identified and their quantification considering the relative area percentage are shown in Tables S1–S5 from Supplementary Materials. It can be observed that the main components of the samples of *C. ladanifer* essential oil were α-pinene (51.93 and 39.11%), followed by viridiflorol (6.40 and 10.21%), bornyl acetate (2.89 and 3.16%), camphene (2.42 and 3.13%), *trans*-pinocarveol (2.15 and 2.22%), and ledol (1.84 and 2.94%). With regard to the *Eucalyptus* essential oils considered, both species showed very high contents of 1,8-cineole (45.02 and 61.26% in *E. globulus* and 73.01 and 73.00% in *E. nitens*), followed by α-pinene (17.70 and 15.89% in *E. globulus* and 11.43 and 10.28% in *E. nitens*), aromadendrene (7.20% but only in the sample corresponding to the first period for *E. globulus*), limonene (4.41 and 5.26% in *E. globulus*, and 2.69 and 2.52% in *E. nitens*), α-terpinyl acetate (4.54 and 4.24%, only in *E. globulus*), *p*-cymene (2.44 and 0.34% in *E. globulus*, and 0.53 and 0.68% in *E. nitens*) and globulol (2.21 and 1.41%, only in *E. globulus*). Concerning the analysis of the *J. communis* essential oils, it can be noticed that both samples showed high contents of sabinene (17.57 and 34.29%) and α-pinene (21.12 and 16.26%), followed by limonene (5.78 and 7.89%), terpinen-4-ol (2.10 and 5.70%), β-myrcene (2.92 and 4.95%), β-phellandrene (2.57 and 3.75%), γ-terpinene (1.41 and 2.56%) and α-thujene (1.60 and 2.51%). On the other hand, thujopsene (12.36%) and *trans*-β-caryophyllene (3.13%) were noticeable in the sample corresponding to the first period. Considering the *Pinus* essential oils analyzed, α-pinene and β-pinene were the main components detected; thus, *P. pinaster* samples had 27.13 and 25.50% of α-pinene and 29.44 and 18.80% of β-pinene, and *P. sylvestris* samples had 45.12 and 51.78% of α-pinene and 8.94 and 19.82% of β-pinene. Both species also showed high contents of β-myrcene (5.97 and 9.45% in *P. pinaster*, and 12.19 and 2.14% in *P. sylvestris*), *trans*-β-caryophyllene (8.31 and 12.06% in *P. pinaster*, and 6.87 and 3.91% in *P. sylvestris*), germacrene D (4.05 and 10.37% in *P. pinaster*, and 6.87 and 4.33% in *P. sylvestris*) and limonene (3.23 and 4.35% in *P. pinaster*, and 1.14 and 3.91% in *P. sylvestris*). Moreover, *P. pinaster* showed 2.11 and 1.70% of longifolene and *P. sylvestris* 3.54 and 3.63% of camphene. Finally, the main components of the samples of *R. officinalis* essential oil were camphor (24.39 and 29.96%), 1,8-cineole (10.71 and 15.73%), and α-pinene (14.61 and 11.75%), followed by camphene (9.70 and 8.00%), β-myrcene (7.96 and 5.62%), limonene (5.96 and 4.52%), β-pinene (4.17 and 4.27%), *trans*-β-caryophyllene (3.39 and 1.93%), 3-octanone (3.21 and 2.42%), bornyl acetate (2.22 and 1.05%), and borneol (2.11 and 1.91%).

### 2.3. Antioxidant Capacity

**Figure 2:**
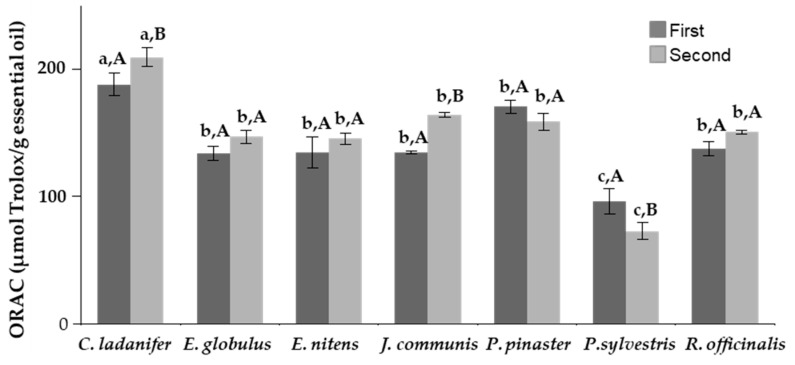
Antioxidant capacity (ORAC) of the essential oils (μmol Trolox/g) from plants collected in the first and second periods. Error bars correspond to the standard deviation of three replicates. (*n* = 3). Different small letters mean significant differences (*p* < 0.05) between all the samples analyzed, whereas different capital letters mean significant differences due to collection period.

Figure 2 represents the antioxidant activity of essential oils measured by ORAC (oxygen radical absorbance capacity) assay and expressed as μmol Trolox per gram of essential oil from plants collected in the first and second periods. All studied essential oils showed antioxidant activity, values ranged from 209.36 to 72.75 μmol Trolox/gram of essential oil. Significant (*p* < 0.05) differences were observed between ORAC values of some studied species and between the two collection periods of *C. ladanifer, J. communis,* and *P. sylvestris*. The essential oil with the highest antioxidant activity was obtained from *C. ladanifer*, in contrast to the lowest activity detected in *P. sylvestris* oil. The essential oils of plants collected in the second period presented higher antioxidant activity (22–8%) than those collected in the first period, except those belonging to genus *Pinus*.

A positive correlation was observed among oxygenated sesquiterpenes and ORAC (r = 0.661, *p* < 0.05).

## 3. Discussion

### 3.1. Essential Oil Extraction

With regard to the distillation tests (Table 1), the significant differences found between the average yields comparing the two collection periods within the same species, with the exception of *Cistus ladanifer* and *Pinus sylvestris*, could be explained by the differences in the plants due to different factors, such as their phenological stage, meteorological conditions or plant age, as other authors have observed in previous studies with these species and others [17,28,29,30,31,32,33].

Different studies carried out with the species considered in this study have been consulted in order to compare the yields obtained. The essential oil yields reported in the literature are shown in Table 3.

As we can observe in Table 1, the species with the highest yield in this study was *E. globulus* (1.59 and 1.93%). However, considering the data shown in Table 3, its yield here is much lower than those obtained by other authors using lab devices, namely, 2.3% [45], 2.7% [41], and 3.9% [44], but higher than the value of 0.5% obtained in a prior study using an industrial device [40]. Considering the values obtained on a laboratory scale (0.7–1.5%) from the literature for *E. nitens* [43,44,46] and the yield obtained in this study (0.45 and 0.57%), it can be observed that the yield from this species is lower than the value corresponding to *E. globulus*.

In the case of *P. pinaster*, the yields obtained in this work were 0.22 and 0.17%, far from the values obtained on a laboratory scale and reported by Rezzoug [52] (0.83%), Mimoune et al. [54] (0.61%), and Amri et al. [53] (0.4%), and close to the figure obtained for plant material collected in central Italy (0.18%) by Macchioni et al. [51].

For *P. sylvestris*, a species with a larger distribution area than *P. pinaster*, many references have been found (Table 3). In most cases, these studies used pine needles for the distillations, and the yields were higher than those obtained in this work. The reported yields reached values higher than 0.8%, compared to 0.15% and 0.18% obtained in the species analyzed here.

*R. officinalis* is a species widely used in the production of essential oil, and there are numerous publications on its composition; however, as indicated above, there are few references regarding its performance in industrial equipment. The reported yields in laboratory equipment were mainly between 1 and 2 (% *w*/*w* d.b.), while in industrial equipment, yields reported in Brazil [66] were between 0.37 and 0.49 (% *v*/*w* d.b.). These numbers are closer to the one obtained in this study (0.5 and 0.44% *w*/*w* d.b.).

In *J. communis*, the yields reported on a laboratory scale in other studies are variable, between 0.05 and 2.43% for foliage (Table 3), and the values obtained in this study (0.37 and 0.31%) are within the limits observed.

Finally, for *C. ladanifer*, the lowest yield values were obtained (0.036 and 0.037%). If these values are compared with the literature consulted (Table 3), it can be observed that they are lower than those obtained on a laboratory scale, between 0.10 and 0.63 (% *w*/*w* d.b.) but similar to the values corresponding to pilot-scale process, between 0.01 and 0.04 (% *v*/*w* d.b.) [39].

### 3.2. Essential Oil Composition

The chemical composition of the essential oils from the samples obtained by steam distillation was determined by GC–MS and GC–FID analyses. The analysis results are shown in Tables S1–S5 from Supplementary Materials.

Comparing the analysis results of *C. ladanifer* essential oil with some works from the literature, the results obtained for α-pinene are within the limits found in samples of commercial essential oils from Spain (48.9–50.4%) [35] and Portugal (29.8–59.5%) [39]. The same trend is observed for bornyl acetate (1.5–3.1% in Spanish essential oils and 2.6–6.1% in Portuguese essential oils), camphene (2.4–5.0% in Spanish essential oils and 2.6–14.7% in Portuguese essential oils), and *trans*-pinocarveol (1.7–2.8% in Spanish essential oils and 1.8–5.9% in Portuguese essential oils). However, higher values of viridiflorol are found in the essential oils analyzed in this work when compared with the limits found in the mentioned Spanish essential oils (1.1–1.7%) and Portuguese essential oils (0.8–1.9%).

On the other hand, considering that the composition of *C. ladanifer* essential oil depends on different factors, such as the origin of the plant, the harvesting season, or the method used to obtain the essential oil [35,39,67,68], compositions of this oil totally different from those obtained in this work have also been found in the literature [35,69,70].

The *E. globulus* essential oils here analyzed showed high contents of 1,8-cineole, followed by α-pinene, aromadendrene, limonene, α-terpinyl acetate, and globulol, listing the compounds according to their decreasing quantities. Comparing these values with those set out by Barbosa et al. [71], it can be observed that 1,8-cineole is always the most abundant compound, with values between 21.4 and 90.0% in essential oils from different countries. The rest of the main compounds analyzed in the studies shown by Barbosa et al. [71] do not follow a common pattern. However, it must be considered that the chemical composition of *Eucalyptus* essential oils depends on the plant stage, and consequently, the harvesting time has significant effects on the chemical composition of this oil [28].

Considering the analyses of the essential oils of *E. globulus* and *E. nitens* obtained, it can be observed that both essential oils are mainly composed of 1,8-cineole and α-pinene, being higher the concentration of 1,8-cineole in the samples corresponding to *E. nitens*. In the work carried out by Dagne et al. [43], 1,8-cineole and *α*-pinene are also the main components in the essential oils of both species, although with similar values of 1,8-cineole.

Concerning the composition of *J. communis* essential oils, some authors have found that the main components from the essential oil obtained from leaves seem to be α-pinene and sabinene [50,72], with values of *α*-pinene between 21.7 and 89.7% and sabinene between 12.1 and 61.9% [72]. Nevertheless, other authors have found that *α*-pinene together with limonene are the most abundant compounds, with concentrations of *α*-pinene between 40.4 and 62.0% and limonene between 4.2 and 10.0% [33]. In the samples analyzed in the present work, sabinene, *α*-pinene, and limonene are the main components, with values next to the lowest limits shown by the above-mentioned authors. Other compounds are also abundant, such as β-myrcene, β-phellandrene, γ-terpinene, α-thujene, thujopsene, and *trans*-β-caryophyllene. They have also been found in *J. communis* essential oils by different authors [33,50,73,74]

The samples of *Pinus pinaster* and *Pinus sylvestris* essential oils here analyzed show that *α*-pinene and β-pinene are the main components of these oils. This aspect has also been observed by Macchioni et al. [51] and Rezzoug [52] in *P. pinaster* essential oils from Italy and France, respectively, and by Koukos et al. [57] in *P. sylvestris* essential oil from Greece. However, other authors have observed that β-pinene is not abundant in some samples of essential oils of *P. pinaster* and *P. sylvestris* from Tunisia [53], Algeria [54,75], Turkey [55], Greece [76], and Lithuania [56,59].

Other components, such as β-myrcene, *trans-*β-caryophyllene, and germacrene D, which are higher than 10% in some of the samples analyzed in the present work, have also been found with a high concentration in the literature [51,57,76].

Finally, concerning *R. officinalis*, two major types of rosemary oil are reported in the literature. The first one with more than 40% of 1,8-cineole and present in Morocco, Tunisia, Turkey, Greece, Yugoslavia, Italy, and France, and the second one with approximately equal ratios of 1,8-cineole, α-pinene, and camphor, and present in France, Spain, Italy, Greece, and Bulgaria [77]. Considering the composition of the two samples analyzed in the present work, where camphor (24.39 and 29.96%), 1,8-cineole (10.71 and 15.73%), and α-pinene (14.61 and 11.75%) are the main components, a greater similarity with the second type can be seen. If these values are compared with those obtained in a different study carried out with *R. officinalis* plants from Spain [78], it can be observed that they are in the same order of magnitude. Furthermore, the essential oils analyzed in the above-mentioned study also showed high contents of camphene, β-pinene, β-caryophyllene, limonene, borneol, and myrcene, as the essential oils analyzed in the present work.

### 3.3. Antioxidant Capacity

As shown in Figure 2, the seven essential oils exhibited antioxidant capacity measured by oxygen radical absorbance capacity assay, as had previously been demonstrated by other authors. Moreover, a correlation between oxygenated sesquiterpenes contents and ORAC values of essential oils was found in the present study.

The essential oil obtained from *C. ladanifer* showed the highest activity by ORAC assay (187.93 and 209.36 μmol Trolox/g essential oil, first and second collection period, respectively). When rockrose essential oil was evaluated by other methods such as the DPPH, β-carotene bleaching, and ABTS assays, also remarkable antioxidant capacity was detected [79,80]. The strong antioxidant capacity, together with the antimicrobial capacity of this essential oil, makes it a promising candidate to use in the food industry.

The antioxidant activity of *Eucalyptus* essential oils evaluated by various testing methods had been reported in earlier studies. The antioxidant activity of *E. globulus* essential oil evaluated by ORAC assay resulted in higher values than the values previously reported by Miguel et al. [81]. However, the antioxidant activity of *E. nitens* has not been investigated in the literature. According to the results presented in Figure 2, the antioxidant activity of the *Eucalyptus* essential oils did not show significant differences between the two studied species (*E. globulus* and *E. nitens*) and the two different collection periods. Neither did Miguel et al. [81] find significant differences among antioxidant activity (ORAC and TEAC methods) of *E. globulus* and some other *Eucalyptus* species. The most antioxidant active components of essential oils were thymol and carvacrol (oxygenated monoterpenes) [27,82,83]. However, essential oils of *E. globulus* and *E. nitens* contain scarcely any amount of these compounds. In fact, the major component found in these *Eucalyptus* essential oils was 1,8-cineole, which showed low antioxidant activity [27,83]. On the other hand, some oxygenated sesquiterpenes analyzed have demonstrated a similar strong antioxidant activity to the oxygenated monoterpenes [27], which could support the relationship between antioxidant activity and oxygenated sesquiterpenes of essential oils deduced from our data. Clearly, the antioxidant activity of essential oils may be due to minor components with important antioxidant activity. Although the chemical complexity of essential oils’ compounds and synergistic and antagonistic effects between these compounds could affect the antioxidant activity [82,84].

In our study, the essential oil of *J. communis* presented similar ORAC values to those of *Eucalyptus* and *P. pinaster* essential oils. Other authors have also observed antioxidant activity of *J. communis* measured by the DPPH method [85]. When the antioxidant capacity of essential oils of different parts of *J. communis* was evaluated, a strong antioxidant activity was detected for berries essential oil of *J. communis* by ORAC (70.5 μmol Trolox/g) [86] and that of the leaves by the DPPH method [19]. As occurs with other essential oils, the main components of *J.* *communis* essential oil (α-pinene, sabinene, and limonene), showed low or even no antioxidant activity determined by different antioxidant evaluation methods [19,27,82]. While *J. communis* essential oil contains a low amount of γ-terpinene, which is a monoterpene with a particularly strong capacity [19,27]. Therefore, the antioxidant activity of *Juniperus* essential oils may also be due to other minor components, such as oxygenated sesquiterpenes.

In relation to *Pinus* essential oils, significant differences were found in antioxidant activity between essential oil of the two *Pinus* species studied in the present work. *P. sylvestris* essential oil showed the lowest activity evaluated by the ORAC assay of all plant materials, and similar results measured by the DPPH assay were found by Kačániová et al. [87]. In contrast, *P. pinaster* essential oil revealed higher activity than *P. sylvestris*. Our results are in accordance with those reported by Tümen et al. [88], in which higher activity for essential oils from *P. pinaster* was also observed, particularly for those obtained of the cones of this Pinus tree.

The antioxidant capacity of *R. officinalis* essential oil demonstrated in our study by the ORAC method was also found by a DPPH free radical scavenging test [65,89,90]. According to Papageorgiou et al. [77], the antioxidant activity of rosemary oil could be due to the synergistic action of minor compounds, instead of the oxygenated sesquiterpenes’ contents. Other authors have identified some oxygenated monoterpenes as responsible for the antioxidant activity of *R. officinalis* [91].

Taking into account the antioxidant capacity of the essential oils obtained from *C. ladanifer*, *E. globulus*, *E. nitens*, *J. communis*, *P. pinaster*, *P. sylvestris*, and *R. officinalis,* these oils could be used as natural antioxidants in the food industry.

## 4. Materials and Methods

### 4.1. Plant Material Collection and Preparation

The plant material (twigs) of each one of the species considered was collected in Spain (central or northern location) in two different periods separated by one or two years, as is shown in Table 4.

Samples were randomly taken from a minimum of 10 plants of a similar age by cutting twigs up to a maximum stem diameter of 30 mm. The biomass of the 10 plants was mixed to obtain samples of 20 kg of green material from each species. A voucher specimen was deposited in the herbarium of the Forest Botany Unit (Forestales building) of the Technical University of Madrid, with reference F-102.

Fresh samples were ground to a size of 30 mm using a shredder (90 kW, slow rotating single-shaft type, 70 r.p.m., SILMISA, Onil, Spain), just before distillation. Subsamples were taken to determine the moisture content in an oven at 105 °C until they reached a constant weight, following the standard ISO 18134-2:2017 [92]. 

### 4.2. Essential Oil Extraction

The freshly ground samples were distilled in a 30 L stainless steel still using steam produced in an electric boiler (ETE, Madrid, Spain). The steam conditions used for the extractions were 15 kg/h of steam with a boiler pressure of 50 kPa. Batch extractions were carried out, with three repetitions of 5 kg each per sample and an extraction duration of 30 min for each batch. Time was measured from the moment the first drop of distillate fell. The temperature inside the still was kept constant at 98 °C. The hydrolate and the essential oil were separated by density using a glass Florentine flask, and the samples were collected in glass flasks. They were then dried using anhydrous sodium sulfate. After filtration, they were weighed and stored in brown bottles at a temperature of 4 °C until further analysis. The oil yield for each sample was calculated as a percentage (*w*/*w*) on a biomass dry weight basis.

**Figure 3:**
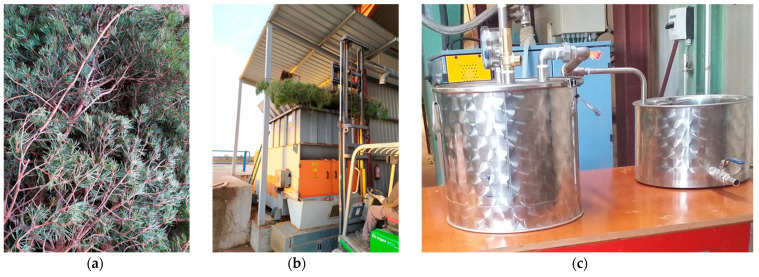
Processes carried out during the pretreatment of *Pinus sylvestris* to obtain essential oil: (**a**) vegetal material collected manually, (**b**) milling, and (**c**) distillation of the milled biomass.

Figure 3 shows the different processes carried out with one of the species used from the manual collection to the distillation.

### 4.3. Essential Oil Analysis

An apparatus Agilent HP 8890/5977 (Agilent Technologies, Santa Clara, CA, USA) was used to identify the essential oil components, and an apparatus Agilent HP 8890 (GC–FID) (Agilent Technologies, Santa Clara, CA, USA) was employed to quantify the identified components. Both of them were equipped with DB-WAX UI-fused silica columns (60 m × 0.25 mm inner diameter, film thickness 0.5 µm) and retention time locking. The column temperature program was 50 °C for 6 min, followed by an increase of 2 °C/min to reach 190 °C; then, a gradient of 4 °C/min to 220 °C was used, and it was maintained for 10 min. Finally, an increase of 4 °C/min was employed to reach 250 °C, which was maintained for 10 min. The carrier gas was helium at a variable flow rate and a head pressure of 30.75 psi. The injection volume was 0.1 µL and split mode injection (ratio 1:100) was used. The temperatures of the injector and detector were 240 °C. GC–MS was performed with the same capillary column, carrier gas, and operating conditions described for GC analysis. Mass spectra were taken over the *m*/*z* range 33–350 with an ionization voltage of 70 eV.

The individual components were quantified using the relative area percentage according to the internal normalization method (ISO 7609:1985 [93]). They were identified by comparison of their Kovats retention index calculated using cochromatographed standard hydrocarbons relative to C6-C30 n-alkanes and mass spectra, with reference samples and those of the computer libraries (NIST 14, Wiley 10, and Chromessence library built through standards injection) and available data in the literature [94].

### 4.4. Antioxidant Activity

Antioxidant activity of essential oils was determined with oxygen radical absorbance capacity (ORAC) methods, as described by Dávalos et al. [95]. Previously, the essential oils were diluted with ethanol (1%). The assay was carried out in 75 mM phosphate buffer (pH 7.4) with the final reaction volume of 200 μL, including 20 μL of the diluted sample, blank or Trolox (used as standard curve 1–8 μM Trolox in each assay), 120 μL of fluorescein solution and 60 μL of AAPH solution, dispensed in a 96-well microplate (NUNC A/S, Roskilde, Denmark) and loaded into a microplate reader (FLUOstar Optima, BMG, Labtech Inc., Durham, NC, USA). The fluorescence was recorded every minute for 104 min (kex = 485 nm, kem = 520 nm). ORAC values were expressed as μmol Trolox equivalents/g of essential oil and were calculated from the regression equation of Trolox concentrations and net area under the fluorescence decay curve, which was generated by FLUOstar Optima software (V2.1 0 R4, BMG Labtech Inc., Durham, NC, USA). All samples were analyzed in triplicate.

### 4.5. Statistical Analysis

Statistical analysis of the data set corresponding to the essential oil yields within the species was performed using Statgraphics Centurion XVII.I version 17.1.06 software package (Statgraphics Technologies, Inc., The Plains, VA, USA). A variance analysis (ANOVA) was used to determine statistically significant differences within the species. A statistically significant difference at a *p*-value of the F-test below 0.05 was considered.

With regard to the antioxidant activity, to identify the statistically significant differences (*p* < 0.05), one-way ANOVA was applied to the obtained analytical data, as well as Duncan’s test. Pearson’s correlation coefficients between the different essential oil components and the ORAC values were calculated using SPSS (Statistical Package for the Social Sciences) 14.0 software package (IBM Corporation, Armonk, NY, USA).

## 5. Conclusions

The exploitation of essential oils obtained from residues of tree and shrub species foliage produced in some forestry enhancement operations could be an incentive to increase forest management while obtaining natural products with added value. In this sense, regarding the yield of the production of essential oil, interesting results are obtained with *E. globulus* and *E. nitens*, considering the tree species, and with *R. officinalis* and *J. communis*, considering the shrub species. Since there are significant differences between the yields obtained with the biomass collected from these species in two different years, more research must be conducted regarding the influence of different factors related to the plant and the weather conditions on the essential oil yield.

In general, the essential oil yields obtained on a pilot scale using the operating conditions described in this work are lower than those performed on a laboratory scale and reported in the literature. Therefore, this study is a step toward the production of essential oils using industrial equipment, although more research must be conducted to optimize the operating conditions during the extraction with the aim of obtaining results that can be extrapolated to an industrial scale.

On the other hand, taking into account the antioxidant activity of the oils studied, and the abundance of the forest residues from which they can be obtained, they could be interesting natural sources of antioxidant additives with potential applications in the food industry as alternatives to synthetic antioxidants. Specifically, oxygenated sesquiterpenes’ contents, one of the minor components of oils, were significantly correlated with ORAC values (*p* < 0.05). However, further investigations are required to determine the antioxidant activity of the active compounds and other biological activities useful in the food industry. The high antioxidant activity by ORAC assay of *Cistus ladanifer* essential oil could increase the interest in this species because, despite its low essential oil yield, its availability in surface area in Spain is, by far, the highest among the studied species.

## Figures and Tables

**Table 1:** Essential oil yields obtained in the steam distillation tests.

Species	Collection Period	Moisture Content (%, w.m.)	Aver. Yield (%, d.b.)	Std. Dev.	*p*-Value of the F-Test
*C. ladanifer*	First	20.2	0.036	0.0023	0.8652
*C. ladanifer*	Second	19.2	0.037	0.0093	
*E. globulus*	First	57.7	1.59	0.021	0.0039
*E. globulus*	Second	77.5	1.93	0.095	
*E. nitens*	First	45.5	0.45	0.0025	0.0001
*E. nitens*	Second	32.8	0.57	0.012	
*J. communis*	First	35.7	0.37	0.011	0.0115
*J. communis*	Second	39.2	0.31	0.020	
*P. pinaster*	First	42.4	0.22	0.0035	0.0001
*P. pinaster*	Second	36.4	0.17	0.0045	
*P. sylvestris*	First	44.3	0.15	0.0046	0.1079
*P. sylvestris*	Second	51.0	0.18	0.026	
*R. officinalis*	First	35.3	0.50	0.010	0.0028
*R. officinalis*	Second	32.6	0.44	0.015	

w.m.: wet matter; d.b.: dry basis; Aver.: average; Std. dev.: standard deviation.

**Table 2:** Component groups of the essential oil samples from plants collected in the first (1) and second (2) periods and analyzed by GC–MS and GC–FID.

Component Groups	Relative Area (%)						
	*C. lad.*	*E. glob.*	*E. nit.*	*J. com.*	*P. pin.*	*P. syl.*	*R. off.*
Monoterpene hydrocarbons (1)	62.47	27.06	16.19	59.19	67.90	74.24	46.01
Monoterpene hydrocarbons (2)	49.46	25.18	14.90	79.58	60.57	84.84	37.75
Oxygenated monoterpenes (1)	14.22	52.19	79.58	3.39	0.75	0.80	43.41
Oxygenated monoterpenes (2)	14.35	68.85	80.46	8.46	1.27	0.48	53.95
Sesquiterpene hydrocarbons (1)	5.38	13.25	1.29	34.20	19.27	19.54	4.75
Sesquiterpene hydrocarbons (2)	6.99	1.75	1.17	8.67	33.53	12.67	4.12
Oxygenated sesquiterpenes (1)	9.47	3.55	0.40	1.65	0.97	0.91	0.03
Oxygenated sesquiterpenes (2)	15.35	2.65	0.54	0.95	0.91	0.48	0.33
Others (1)	2.77	0.42	1.30	0.52	9.88	0.12	4.17
Others (2)	3.35	0.25	1.61	0.71	2.73	0.72	2.98
Total identified (1)	94.31	96.47	98.76	98.95	98.77	95.61	98.37
Total identified (2)	89.50	98.68	98.68	98.37	99.01	99.19	99.13

*C. lad.: Cistus ladnifer; E. glob.: Eucalyptus globulus; E. nit.: Eucalyptus nitens; J. com.: Juniperus communis; P. pin.: Pinus pinaster; P. syl.: Pinus sylvestris; R. off.: Rosmarinus officinalis*.

**Table 3:** Essential oil yields reported in previous works for the species studied.

Species	Ref.	Collection Months	Origin	Plant Part	Distil. Method	Distil. Time (h)	Sample Weight	Yield (%*w*/*w* d.b.)	Yield (%*v*/*w* d.b.)
C. lad.	[34]	n.a.	Portugal	twigs	SD	n.a.	50 g	0.33	-
	[35]	July–August	Portugal	twigs	HD Clev	2	20 g	0.25	-
	[36]	May	France	twigs	SD	2	100 g	0.10	-
	[37]	July	Portugal	twigs	SDE	1	15 g	0.15	-
	[38]	July	Portugal	leaves	HD Clev	3	100 g	0.63	-
	[39]	March	Portugal	twigs	SD	1.5	100 kg	-	0.01
	[39]	August	Portugal	twigs	SD	1.5	100 kg	-	0.04
	[39]	August	Portugal	twigs	HD	3	270 g	-	0.15
E. glob.	[40]	n.a.	Algeria	twigs	SD	n.a.	600 kg	0.5	-
	[41]	n.a.	Morocco	leaves	HD Clev	2	n.a.	2.7	-
	[42]	March	Uganda	leaves	HD Clev	n.a.	n.a.	-	0.2
	[43]	n.a.	Ethiopia	leaves	HD Clev	3	n.a.	1.1	-
	[44]	March	Australia	leaves	Glass dist	6	100 g	3.9	-
	[45]	n.a.	Morocco	leaves	SD	n.a.	n.a.	2.3	-
E. nit.	[43]	n.a.	Ethiopia	leaves	HD Clev	3	n.a.	1.4	-
	[44]	March	Australia	leaves	Glass dist	6	100 g	0.75	-
	[46]	November	Australia	leaves	Glass dist	6	150 g	0.7–1.5	-
J. com.	[17]	February	Estonia	leaves	SDE	2	10 g	0.70	-
	[47]	October–December	Sardinia	leaves	SD Clev	n.a.	100 g	-	0.19
	[33]	May–April	Estonia	branches	HD Clev	1.5	20 g	0.05–0.70	-
	[48]	April–December	India	leaves	SD Clev	3	1000 g	0.70	-
	[49]	May–November	Iran	twigs	HD Clev	3.5	30 g	-	2.43
	[50]	Summer	Bulgaria	leaves	HD Clev	2	50 g	-	0.30–0.60
	[50]	Summer	Serbia	leaves	HD Clev	2	50 g	-	0.53–0.86
P. pin.	[51]	February	Italy	twigs	HD Clev	2	n.a.	0.18	-
	[52]	n.a.	France	leaves	HD Clev	2	50 g	0.82	-
	[53]	August	Tunisia	needles	HD Clev	3	100 g	0.4	-
	[54]	March	Algeria	needles	HD Clev	4	100 g	0.61	-
P. syl.	[55]	September	Turkey	cones	HD Clev	3	100 g		0.13
	[56]	July	Lithuania	leaves	HD Clev	2	50 g	0.25	-
	[57]	April	Greece	twigs	HD	3	80 g	0.52	-
	[58]	n.a.	Turkey	leaves	HD Clev	3	n.a.	0.22–0.82	-
	[59]	July	Lithuania	leaves	HD Clev	2	20 g	-	1
	[60]	January	Lithuania	leaves	HD Clev	2	70 g	0.43–0.64	-
R. off.	[42]	March	Uganda	leaves	HD Clev	n.a.	n.a.	-	1
	[61]	n.a.	Spain	twigs	HD Clev	2	100 g	1.88	-
	[62]	n.a.	Spain	twigs	SD	n.a.	n.a.	1.1	-
	[41]	n.a.	Morocco	leaves	HD Clev	2	n.a.	1.8	-
	[63]	October–May	Algeria	twigs	HD	n.a.	n.a.	0.64–1.07	-
	[64]	January–December	Italy	twigs	SD	n.a.	900 g	0.055–0.77	-
	[65]	November–December	Pakistan	leaves	HD Clev	3	n.a.	0.93	-
	[66]	n.a.	Brazil	twigs	SD	1.5	60–310 kg	-	0.37–0.49

Ref.: reference; Distil.: distillation; w: weight; v: volume; d.b.; dry basis; SDE: simultaneous distillation extraction; SD: steam distillation; HD: hydrodistillation; Clev: Clevenger; Glass dist: glass distiller; n.a.: not available.

**Table 4:** Location and date of the plant material collection.

Species	Coordinates (WGS84 Google)	1st Collection Date	2nd Collection Date
*Cistus ladanifer*	X: −2.979643 Y: 41.09917	September 2018	October 2019
*Eucalyptus globulus*	X: −5.31016776 Y: 43.5112467	July 2018	June 2020
*Eucalyptus nitens*	X: −7.3083651 Y: 41.9822278	July 2018	April 2020
*Juniperus communis*	X: −2.463912 Y: 42.004695	May 2018	June 2020
*Pinus pinaster*	X: −2.490868 Y: 41.601647	July 2018	April 2020
*Pinus sylvestris*	X: −2.478987 Y: 42.033522	July 2018	April 2020
*Rosmarinus officinalis*	X: −1.988602 Y: 41.562386	July 2018	April 2020

## Supplementary Materials

The following are available online, Table S1: Analysis of the essential oils by GC–MS and GC–FID: monoterpene hydrocarbons, Table S2: Analysis of the essential oils by GC–MS and GC–FID: oxygenated monoterpenes, Table S3: Analysis of the essential oils by GC–MS and GC–FID: sesquiterpene hydrocarbons, Table S4: Analysis of the essential oils by GC–MS and GC–FID: oxygenated sesquiterpenes, Table S5: Analysis of the essential oils by GC–MS and GC–FID: others.

## Sample Availability

The data leading to the results of this work are available from the authors upon reasonable request.

## References

[1] LUCAS (2018). Land Use and Cover Area frame Survey. EUROSTAT. https://ec.europa.eu/eurostat/web/lucas/data/primary-data.

[2] Bongers F., Olmo M., Lopez-Iglesias B., Anten N., Villar R. (2017). Drought responses, phenotypic plasticity and survival of Mediterranean species in two different microclimatic sites. Plant Biol..

[3] Rundel P., Arroyo M., Cowling R., Keeley J., Lamont B., Pausas J., Vargas P. (2018). Fire and Plant Diversification in Mediterranean-Climate Regions. Front. Plant Sci..

[4] Francos M., Úbeda X., Pereira P., Alcañiz M. (2018). Long-term impact of wildfire on soils exposed to different fire severities. A case study in Cadiretes Massif (NE Iberian Peninsula). Sci. Total Environ..

[5] Fowler C. (2003). Human health impacts of forest fires in the Southern United States: A literature review. J. Ecol. Antropol..

[6] Liu Z., Wimberly M. (2016). Direct and indirect effects of climate change on projected future fire regimes in the western United States. Sci. Total Environ..

[7] Bussotti F., Ferrini F., Pollastrini M., Fini A. (2014). The challenge of Mediterranean sclerophyllous vegetation under climate change: From acclimation to adaptation. Environ. Exp. Bot..

[8] Verkerk P., de Arano I., Palahi M. (2018). The bio-economy as an opportunity to tackle wildfires in Mediterranean forest ecosystems. For. Pol. Econ..

[9] Nazzaro F., De Martino L., Fratianni F., De Feo V., Preedy V.R., Watson R.R. (2020). Essential oils from Mediterranean aromatic plants. The Mediterranean Diet. An Evidence-Based Approach.

[10] Sharma S., Barkauskaite S., Jaiswal A., Jaiswal S. (2021). Essential oils as additives in active food packaging. Food Chem..

[11] Pateiro M., Munekata P.E.S., Sant′Ana A.S., Dominguez R., Rodriguez-Lazaro D., Lorenzo J.M. (2021). Application of essential oils as antimicrobial agents against spoilage and pathogenic microorganisms in meat products. Int. J. Food Microbiol..

[12] GBIF GBIF Secretariat (2019). GBIF Backbone Taxonomy. Checklist Dataset. https://www.gbif.org/es/species/6.

[13] MFE25. Mapa Forestal de España (2018). MAPAMA. Dirección General de Desarrollo Rural. https://www.miteco.gob.es/es/cartografia-y-sig/ide/descargas/biodiversidad/mfe.aspx.

[14] MFE200. Mapa Forestal de España (1997). Dirección General de Medio Natural y Política Forestal. Ministerio de Medio Ambiente, y Medio Rural y Marino. https://www.miteco.gob.es/es/biodiversidad/servicios/banco-datos-naturaleza/informacion-disponible/mfe200_descargas.aspx/.

[15] Hernández M., Sotomayor J., Jordán M., Preedy V. (2016). Rosemary (*Rosmarinus officinalis* L.) oils. Essential Oils in Food Preservation, Flavor and Safety.

[16] Jeronimo E., Cachucho L., Soldado D., Guerreiro O., Bessa R., Alves S. (2020). Fatty Acid Content and Composition of the Morphological Fractions of *Cistus Ladanifer* L. and Its Seasonal Variation. Molecules.

[17] Orav A., Koel M., Kailas T., Muurisepp M., Kaljurand M. (2010). Comparative analysis of the composition of essential oils and supercritical carbon dioxide extracts from the berries and needles of Estonian juniper (*Juniperus communis* L.). Procedia Chem..

[18] Said Z., Haddadi-Guemghar H., Boulekbache-Makhlouf L., Rigou P., Remini H., Adjaoud A., Khoudja N., Madani K. (2016). Essential oils composition, antibacterial and antioxidant activities of hydrodistillated extract of Eucalyptus globulus fruits. Ind. Crops. Prod..

[19] Emami A., Javadi B., Hassanzadeh M. (2007). Antioxidant activity of the essential oils of different parts of Juniperus communis subsp hemisphaerica and Juniperus oblonga. Pharm Biol..

[20] Höferl M., Stoilova I., Schmidt E., Wanner J., Jirovetz L., Trifonova D., Krastev L., Krastanov A. (2014). Chemical Composition and Antioxidant Properties of Juniper Berry (*Juniperus communis* L.) Essential Oil. Action of the Essential Oil on the Antioxidant Protection of Saccharomyces cerevisiae Model Organism. Antioxidants.

[21] Arnal-Schnebelen B., Hadji-Minaglow F., Peroteau J., Ribeyre F., de Billerbeck V. (2004). Essential oils in infectious gynaecological disease: A statistical study of 658 cases. Int. J. Aromather..

[22] Verdeguer M., Blázquez M., Boira H. (2012). Chemical composition and herbicidal activity of the essential oil from a *Cistus ladanifer* L. population from Spain. Nat. Prod. Res..

[23] Lü J.M., Lin P.H., Yao Q., Chen C. (2010). Chemical and molecular mechanisms of antioxidants: Experimental approaches and model systems. J. Cell Mol. Med..

[24] Liguori I., Russo G., Curcio F., Bulli G., Aran L., Della-Morte D., Gargiulo G., Testa G., Cacciatore F., Bonaduce D. (2018). Oxidative stress, aging, and diseases. Clin. Interv. Aging..

[25] Luo J., Mills K., le Cessie S., Noordam R., van Heemst D. (2020). Ageing, age-related diseases and oxidative stress: What to do next?. Ageing. Res. Rev..

[26] Teixeira B., Marques A., Ramos C., Neng N.R., Nogueira J.M.F., Saraiva J.A., Nunes M.L. (2013). Chemical composition and antibacterial and antioxidant properties of commercial essential oils. Ind. Crops. Prod..

[27] Ruberto G., Baratta M.T. (2000). Antioxidant activity of selected essential oil components in two lipid model systems. Food Chem..

[28] Salem N., Kefi S., Tabben O., Ayed A., Jallouli S., Feres N., Hammami M., Khammassi S., Hrigua I., Nefisi S. (2018). Variation in chemical composition of *Eucalyptus globulus* essential oil under phenological stages and evidence synergism with antimicrobial standards. Ind. Crops. Prod..

[29] Daghbouche S., Ammar I., Rekik D., Djazouli Z., Zebib B., Merah O. (2020). Effect of phenological stages on essential oil composition of *Cytisus triflorus* L’Her. J. King Saud Univ. Sci..

[30] Alipour M., Saharkhiz M. (2016). Phytotoxic activity and variation in essential oil content and composition of Rosemary (*Rosmarinus officinalis* L.) during different phenological growth stages. Biocatal. Agric. Biotechnol..

[31] Vaiciulyte V., Butkiene R., Loziene K. (2016). Effects of meteorological conditions and plant growth stage on the accumulation of carvacrol and its precursors in Thymus pulegioides. Phytochemistry.

[32] Usano-Alemany J., Pala-Paul J., Herraiz-Penalver D. (2016). Essential oil yields and qualities of different clonal lines of *Salvia lavandulifolia* monitored in Spain over four years of cultivation. Ind. Crops. Prod..

[33] Raal A., Kanut M., Orav A. (2010). Annual Variation of Yield and Composition of the Essential Oil of Common Juniper (*Juniperus communis* L.) Branches from Estonia. Balt. For..

[34] Mendes B.S., Gonçalves M.M.B.P. (2013). Composition and functional properties of essential oils extracted from forest waste biomass. Studia Suplemento temático.

[35] Gomes P., Mata V., Rodrigues A. (2005). Characterization of the Portuguese-grown Cistus ladanifer essential oil. J. Essent. Oil Res..

[36] Robles C., Bousquet-Melou A., Garzino S., Bonin G. (2003). Comparison of essential oil composition of two varieties of Cistus ladanifer. Biochem. Syst. Ecol..

[37] Teixeira S., Mendes A., Alves A., Santos L. (2007). Simultaneous distillation-extraction of high-value volatile compounds from *Cistus ladanifer* L.. Anal. Chim. Acta.

[38] Guimaraes R., Sousa M., Ferreira I. (2010). Contribution of essential oils and phenolics to the antioxidant properties of aromatic plants. Ind. Crops Prod..

[39] Tavares C., Martins A., Faleiro M., Miguel M., Duarte L., Gameiro J., Roseiro L., Figueiredo A. (2020). Bioproducts from forest biomass: Essential oils and hydrolates from wastes of *Cupressus lusitanica* Mill. and *Cistus ladanifer* L.. Ind. Crops Prod..

[40] Boukhatem M., Amine F., Kameli A., Saidi F., Walid K., Mohamed S. (2014). Quality assessment of the essential oil from *Eucalyptus globulus* Labill of Blida (Algeria) origin. ILCPA.

[41] Ait-Ouazzou A., Loran S., Bakkali M., Laglaoui A., Rota C., Herrera A., Pagan R., Conchello P. (2011). Chemical composition and antimicrobial activity of essential oils of Thymus algeriensis, Eucalyptus globulus and Rosmarinus officinalis from Morocco. J. Sci. Food Agric..

[42] Cuéllar Cuéllar A., Hussein Yunus R. (2009). Evaluation of the yield and the antimicrobial activity of the essential oils from: *Eucalyptus globulus, Cymbopogon citratus* and *Rosmarinus officinalis* in Mbarara district (Uganda). Rev. Colomb. Cienc. Anim..

[43] Dagne E., Bisrat D., Alemayehu M., Worku T. (2000). Essential oils of twelve Eucalyptus species from Ethiopia. J. Essent. Oil Res..

[44] Li H., Madden J. (1995). Analysis of leaf oils from a Eucalyptus species trial. Biochem. Syst. Ecol..

[45] Zrira S., Benjilali B. (1996). Seasonal changes in the volatile oil and cineole contents of five *Eucalyptus* species growing in Morocco. J. Essent. Oil Res..

[46] Li H., Madden J., Davies N. (1994). Variation in leaf oils of *Eucalyptus nitens* and *E. Denticulata*. Biochem. Syst. Ecol..

[47] Angioni A., Barra A., Russo M., Coroneo V., Dessi S., Cabras P. (2003). Chemical composition of the essential oils of *Juniperus* from ripe and unripe berries and leaves and their antimicrobial activity. J. Agric. Food Chem..

[48] Koundal R., Kumar A., Thakur S., Agnihotri V., Chand G., Singh R. (2015). Seasonal variation in phytochemicals of essential oil from *Juniperus communis* needles in western Himalaya. J. Essent. Oil Res..

[49] Rostaefar A., Hassani A., Sefidkon F. (2017). Seasonal variations of essential oil content and composition in male and female plants of Juniperus communis L. ssp hemisphaerica growing wild in Iran. J. Essent. Oil Res..

[50] Radoukova T., Zheljazkov V., Semerdjieva I., Dincheva I., Stoyanova A., Kacaniova M., Markovic T., Radanovic D., Astatkie T., Salamon I. (2018). Differences in essential oil yield, composition, and bioactivity of three juniper species from Eastern Europe. Ind. Crops Prod..

[51] Macchioni F., Cioni P., Flamini G., Morelli I., Maccioni S., Ansaldi M. (2003). Chemical composition of essential oils from needles, branches and cones of Pinus pinea, P-halepensis, P-pinaster and P. nigra from central Italy. Flavour. Fragr. J..

[52] Rezzoug S. (2009). Optimization of Steam Extraction of Oil from Maritime Pine Needles. J. Wood Chem. Technol..

[53] Amri I., Hanana M., Gargouri S., Jamoussi B., Hamrouni L. (2013). Comparative study of two coniferous species (*Pinus pinaster* Aiton and *Cupressus sempervirens* L. var. dupreziana [A. Camus] Silba) essential oils: Chemical composition and biological activity. Chil. J. Agric. Res..

[54] Mimoune N., Mimoune D., Yataghene A. (2013). Chemical composition and antimicrobial activity of the essential oils of P*inus pinaster*. J. Coast. Life Med..

[55] Tumen I., Hafizoglu H., Kilic A., Donmez I., Sivrikaya H., Reunanen M. (2010). Yields and Constituents of Essential Oil from Cones of *Pinaceae* spp. Natively Grown in Turkey. Molecules.

[56] Judzentiene A., Kupcinskiene E. (2008). Chemical composition on essential oils from needles of Pinus sylvestris L. grown in northern Lithuania. J. Essent. Oil Res..

[57] Koukos P., Papadopoulou K., Papagiannopoulos A., Patiaka D. (2001). Essential oils of the twigs of some conifers grown in Greece. Holz Als Roh-Und Werkstoff.

[58] Ustun O., Sezik E., Kurkcuoglu M., Baser K. (2006). Study of the essential oil composition of Pinus sylvestris from Turkey. Chem. Nat. Compd..

[59] Venskutonis P., Vyskupaityte K., Plausinaitis R. (2000). Composition of essential oils of Pinus sylvestris L. from different locations of Lithuania. J. Essent. Oil Res..

[60] Labokas J., Loziene K., Jureviciute R. (2017). Preconditions for industrial use of foliage as felling by-product of Scots pine for essential oil production. Ind. Crops Prod..

[61] Varela F., Navarrete P., Cristobal R., Fanlo M., Melero R., Sotomayor J., Jordan M., Cabot P., de Ron D., Calvo R. (2009). Variability in the chemical composition of wild *Rosmarinus officinalis* L.. Int. Med. Aromat. Plants Conf. Culin. Herbs.

[62] Guillén M., Cabo N., Burillo J. (1996). Characterisation of the essential oils of some cultivated aromatic plants of industrial interest. J. Sci. Food Agric..

[63] Boutekedjiret C., Belabbes R., Bentahar F., Bressiere J. (1999). Study of *Rosmarinus officinalis* L. essential oil yield and composition as a function of the plant life cycle. J. Essent. Oil Res..

[64] Serralutzu F., Stangoni A., Amadou B., Tijan D., Re G., Marceddu S., Dore A., Bullitta S. (2020). Essential oil composition and yield of a *Rosmarinus officinalis* L. natural population with an extended flowering season in a coastal Mediterranean environment and perspectives for exploitations. Genet. Resour. Crop Evol..

[65] Hussain A., Anwar F., Chatha S., Jabbar A., Mahboob S., Nigam P. (2010). *Rosmarinus* officinalis essential oil: Antiproliferative, antioxidant and antibacterial activities. Braz. J. Microbiol..

[66] Atti-Santos A., Rossato M., Pauletti G., Rota L., Rech J., Pansera M., Agostini F., Serafini L., Moyne P. (2005). Physico-chemical evaluation of *Rosmarinus officinalis* L. essential oils. Braz. Archz. Biol. Technol..

[67] Barrajón-Catalán E., Tomás-Menor L., Morales-Soto A., Martí Bruñá N., Saura López D., Segura-Carretero A., Micol V., Preedy V.R. (2015). Rockroses (C*istus* sp.) oils. Essential Oils in Food Preservation, Flavor and Safety.

[68] Frazao D., Raimundo J., Domingues J., Quintela-Sabaris C., Goncalves J., Delgado F. (2018). *Cistus ladanifer* (Cistaceae): A natural resource in Mediterranean-type ecosystems. Planta.

[69] Viuda-Martos M., Sendra E., Perez-Alvarez J., Fernandez-Lopez J., Amensour M., Abrini J. (2011). Identification of flavonoid content and chemical composition of the essential oils of Moroccan herbs: Myrtle (*Myrtus communis* L.), rockrose (*Cistus ladanifer* L.) and Montpellier cistus (*Cistus monspeliensis* L.). J. Essent. Oil Res..

[70] Benali T., Bouyahya A., Habbadi K., Zengin G., Khabbach A., Achbani E., Hammani K. (2020). Chemical composition and antibacterial activity of the essential oil and extracts of Cistus ladaniferus subsp. ladanifer and Mentha suaveolens against phytopathogenic bacteria and their ecofriendly management of phytopathogenic bacteria. Biocatal. Agric. Biotechnol..

[71] Barbosa L., Filomeno C., Teixeira R. (2016). Chemical Variability and Biological Activities of Eucalyptus spp. Essential Oils. Molecules.

[72] Ložienė K., Venskutonis P., Preedy V.R. (2016). Juniper (*Juniperus communis* L.) oils. Essential Oils in Food Preservation, Flavor and Safety.

[73] Adams R., Beauchamp P., Dev V., Bathala R. (2010). The Leaf Essential Oils of *Juniperus communis* L. Varieties in North America and the NMR and MS Data for Isoabienol. J. Essent. Oil Res..

[74] Hadaruga N., Branic A., Hadaruga D., Gruia A., Plesa C., Costescu C., Ardelean A., Lupea A. (2011). Comparative study of *Juniperus communis* and *Juniperus virginiana* essential oils: TLC and GC analysis. JPC-J. Planar. Chromat..

[75] Dob T., Berramdane T., Chelghoum C. (2005). Analysis of essential oil from the needles of *Pinus pinaster* growing in Algeria. Chem. Nat. Compd..

[76] Petrakis P., Tsitsimpikou C., Tzakou O., Couladis M., Vagias C., Roussis V. (2001). Needle volatiles from five Pinus species growing in Greece. Flavour. Fragr. J..

[77] Papageorgiou V., Gardeli C., Mallouchos A., Papaioannou M., Komaitis M. (2008). Variation of the chemical profile and antioxidant behavior of *Rosmarinus officinalis* L. and Salvia fruticosa Miller grown in Greece. J. Agric. Food Chem..

[78] Salido S., Altarejos J., Nogueras M., Sanchez A., Luque P. (2003). Chemical composition and seasonal variations of rosemary oil from southern Spain. J. Essent. Oil Res..

[79] Luís Â., Ramos A., Domingues F. (2020). Pullulan Films Containing Rockrose Essential Oil for Potential Food Packaging Applications. Antibiotics.

[80] Upadhyay N., Singh V., Dwivedy A., Das S., Chaudhari A., Dubey N. (2018). Cistus ladanifer L. essential oil as a plant based preservative against molds infesting oil seeds, aflatoxin B 1 secretion, oxidative deterioration and methylglyoxal biosynthesis. LWT.

[81] Miguel M.G., Gago C., Antunes M.D., Lagoas S., Faleiro M.L., Megías C., Cortés-Giraldo I., Vioque J., Figueiredo A.C. (2018). Antibacterial, antioxidant, and antiproliferative activities of *Corymbia citriodora* and the essential oils of eight Eucalyptus species. Medicines.

[82] Bentayeb K., Vera P., Rubio C., Nerín C. (2014). The additive properties of Oxygen Radical Absorbance Capacity (ORAC) assay: The case of essential oils. Food Chem..

[83] Lee K.-G., Shibamoto T. (2001). Antioxidant activities of volatile components isolated from Eucalyptus species. J. Sci. Food Agric..

[84] Ben Marzoug H.N., Bouajila J., Ennajar M., Lebrihi A., Mathieu F., Couderc F., Abderraba M., Romdhane M. (2010). Eucalyptus (gracilis, oleosa, salubris, and salmonophloia) essential oils: Their chemical composition and antioxidant and antimicrobial activities. J. Med. Food.

[85] Elshafie H.S., Caputo L., De Martino L., Gruľová D., Zheljazkov V.Z., De Feo V., Camele I. (2020). Biological investigations of essential oils extracted from three *Juniperus* species and evaluation of their antimicrobial, antioxidant and cytotoxic activities. J. Appl. Microbiol..

[86] Zheljazkov V.D., Kacaniova M., Dincheva I., Radoukova T., Semerdjieva I.B., Astatkie T., Schlegel V. (2018). Essential oil composition, antioxidant and antimicrobial activity of the galbuli of six juniper species. Ind. Crops Prod..

[87] Kačániová M., Vukovič N., Horská E., Salamon I., Bobková A., Hleba L., Fiskelová M., Vatľák A., Petrová J., Bobko M. (2014). Antibacterial activity against Clostridium genus and antiradical activity of the essential oils from different origin. J. Environ. Sci. Health B.

[88] Tümen İ., Akkol E.K., Taştan H., Süntar I., Kurtca M. (2018). Research on the antioxidant, wound healing, and anti-inflammatory activities and the phytochemical composition of maritime pine (Pinus pinaster Ait). J. Ethnopharmacol..

[89] Viuda-Martos M., Ruiz Navajas Y., Sánchez Zapata E., Fernández-López J., Pérez-Álvarez J.A. (2010). Antioxidant activity of essential oils of five spice plants widely used in a Mediterranean diet. Flavour. Fragr. J..

[90] Rašković A., Milanović I., Pavlović N., Ćebović T., Vukmirović S., Mikov M. (2014). Antioxidant activity of rosemary (Rosmarinus officinalis L.) essential oil and its hepatoprotective potential. BMC Complement. Altern. Med..

[91] Mezza G.N., Borgarello A.V., Grosso N.R., Fernandez H., Pramparo M.C., Gayol M.F. (2018). Antioxidant activity of rosemary essential oil fractions obtained by molecular distillation and their effect on oxidative stability of sunflower oil. Food Chem..

[92] ISO 18134–2:2017 Solid Biofuels–Determination of Moisture Content–Oven Dried Method–Part 2: Total Moisture–Simplified Method.

[93] ISO 7609:1985 Analysis by Gas Chromatography on Capillary Columns–General Method.

[94] Adams R. (2007). Identification of Essential Oil Components by Gras Chromatography/Mass Spectrometry.

[95] Dávalos A., Gómez-Cordovés C., Bartolomé B. (2004). Extending applicability of the oxygen radical absorbance capacity (ORAC-fluorescein) assay. J. Agric. Food Chem..

