# Supplementary material for: Essential Oils from Residual Foliage of Forest Tree and Shrub Species: Yield and Antioxidant Capacity
Source: Molecules. 2021 May 28;26(11):3257. doi: 10.3390/molecules26113257 (PMC8198416; doi:10.3390/molecules26113257)
Supplement: Supplementary file 1 [file molecules-26-03257-s001.zip › molecules-1232422-supplementary.pdf]

Table S1. Analysis of the essential oils by GC–MS and GC–FID: monoterpene hydrocarbons.

Component	RI_{cal}	RI_{ref}	C. lad.[1]	C. lad.[2]	E. glob.[1]	E. glob.[2]	E. nit.[1]	E. nit.[2]	J. com.[1]	J. com.[2]	P. pin.[1]	P. pin.[2]	P. syl.[1]	P. syl.[2]	R. off.[1]	R. off.[2]
Trans-Pinane	940		0.03	0.02												
Tricyclene	1001	1012	0.33	0.44			0.04	0.01	0.06	0.05	0.04	0.04	0.88	0.84	0.39	0.31
α-Pinene	1021	1025	51.93	39.11	17.70	15.89	11.43	10.28	21.12	16.26	27.13	25.50	45.12	51.78	14.61	11.75
α-Thujene	1024	1026	0.26	0.18	0.15	0.05	0.02	0.03	1.60	2.51	0.01	0.01		0.03	0.11	0.11
α-Fenchene	1056	1061	0.06	0.06	0.01	0.01	0.02	0.02	0.06	0.04	0.01	0.01	0.01	0.01	0.08	0.08
Camphene	1061	1068	2.42	3.13	0.03	0.09	0.26	0.1	0.16	0.12	0.35	0.29	3.54	3.63	9.70	8.00
β-Pinene	1106	1110	0.68	0.78	0.54	0.61	0.49	0.27	1.49	1.17	29.44	18.80	8.94	19.82	4.17	4.27
Sabinene	1118	1122	0.68	0.49					17.57	34.29	0.05	0.03	0.15	0.17	0.08	0.08
Verbenene	1123	1123	0.63	0.51	0.01	0.01	0.02	0.04	0.05	0.02	0.02	0.04	0.04	0.03	0.11	0.14
δ-4-Carene	2005	1128								0.18						
δ-3-Carene	1147	1147							0.72	0.37	0.01	0.01		0.02	0.01	0.03
β-Myrcene	1161	1161	0.05	0.06	0.5	0.73	0.54	0.08	2.92	4.95	5.97	9.45	12.19	2.14	7.96	5.62
α-Phellandrene	1164	1167	0.06	0.07	0.62	0.26	0.09	0.47	0.67	0.92	0.01	0.02	0.02	0.12	0.15	0.21
p-Mentha-1(7),8-diene	1169	1172			0.01	0.01	0.01	0.01							0.01	0.02
3,7,7-Trimethyl-1,3,5-cycloheptatriene	1177	-							0.01							
α-Terpinene	1179	1178	0.62	0.49	0.04	0.09			0.74	1.4	0.01	0.02	0.02	0.03	0.16	0.28
Menthatriene Isomer	1197	-	0.06	0.07												
Limonene	1199	1198	1.57	1.31	4.41	5.26	2.69	2.52	5.78	7.89	3.23	4.35	1.14	3.91	5.96	4.52
β-Phellandrene	1212	1209	0.44	0.44	0.07	0.15		0.02	2.57	3.75	0.98	0.77	0.68	1.12	0.37	0.35
Cis-β-Ocimene	1234	1234			0.08	0.35				0.01	0.01	0.01	0.03	0.02	0.07	0.06
γ-Terpinene	1247	1245	1.18	0.93	0.23	1.01	0.03	0.07	1.41	2.56	0.02	0.03	0.04	0.07	0.29	0.35
Trans-β-Ocimene	1251	1250						0.08	0.07		0.25	0.26	1.27	0.64	0.02	0.01
Para-Cymene	1272	1270	1.1	0.97	2.44	0.34	0.53	0.68	0.85	1.06			0.05	0.06	1.28	1.15
α-Terpinolene	1285	1282	0.28	0.24	0.19	0.31		0.19	1.29	2.03	0.35	0.91	0.12	0.39	0.44	0.36

α-p-Dimethylstyrene	1441	1438	0.09	0.16	0.03	0.01	0.02	0.03	0.05		0.01	0.02		0.01	0.04	0.05

RI_{cal}: retention index obtained in a DB-WAX column; RI_{ref}: retention index based on bibliography; [1]: first sampling period; [2]: second sampling period; C. lad.: *Cistus ladnifer*; E. glob.: *Eucalyptus globulus*; J. com.: *Juniperus communis*; P. pin.: *Pinus pinaster*; P. syl.: *Pinus sylvestris*; R. off.: *Rosmarinus officinalis*.

Table S2. Analysis of the essential oils by GC–MS and GC–FID: oxygenated monoterpenes.

Component	RI_{cal}	RI_{ref}	C. lad.[1]	C. lad.[2]	E. glob.[1]	E. glob.[2]	E. nit.[1]	E. nit.[2]	J. com.[1]	J. com.[2]	P. pin.[1]	P. pin.[2]	P. syl.[1]	P. syl.[2]	R. off.[1]	R. off.[2]
2,3-Dehydro-1,8-cineole	1194	1192					0.01	0.01								
1,8-Cineole	1209	1211	0.21	0.2	45.02	61.26	73.01	73.00			0.01	0.02		0.05	10.71	15.73
Isoamyl Isovalerate	1294	1294			0.05	0.25										
2,2,6-Trimethylcyclohexanone	1323	1333	0.93	0.74												
Pinol	1330	-	0.04	0.03	0.03											
Cis-Rose Oxide	1357	1358	0.2	0.17												
Trans-Rose Oxide	1373	-	0.09	0.07												
Fenchone	1405	1399													0.02	0.29
Perillene	1420	1425								0.01	0.01	0.01				
α-Thujone	1437	1424								0.01						
Campholenal isomer	1449	-	0.27	0.25		0.01										
Cis-Linalool Oxide (Furanoid)	1453	1453					0.01	0.01								
β-Thujone	1468	-							0.02	0.07						
Trans-Sabinene Hydrate	1470	1467	0.14	0.11					0.09	0.41						0.08
Nerol Oxide	1476	1469	0.15	0.14												
Trans-Linalool Oxide (Furanoid)	1474	1478					0.01	0.03								
α Campholenal	1496	1496	1.37	1.24	0.07	0.01	0.03	0.04	0.02	0.03	0.02	0.06		0.03	0.01	0.03
Chrysanthenone	1517	1507													0.11	0.14
Pinocamphone	1529	1523									0.03	0.1				
Camphor	1525	1515	0.13	0.17			0.03	0.19	0.03						24.39	29.96
Linalool	1544	1543	0.07	0.09		0.08	0.05	0.01	0.06						1.08	1.26

Isopinocamphone	1556	1544	0.65	0.6										0.04	0.23	0.26
Cis-Sabinene Hydrate	1552	1549								0.2						
Linalyl Acetate	1558	1554							0.01		0.14	0.16				
Trans-Para-Menth-2-en-1-ol	1633	1641					0.01	0.01	0.05	0.25					0.02	0.02
Terpinen-1-ol	1574	1573													0.03	0.02
Isopulegol	1581	-													0.11	0.13
Pinocarvone	1582	-	1.44	1.35	0.29	0.03	0.77	0.84			0.02	0.03		0.02	0.05	0.06
Fenchol <endo>	1586	1570			0.12		0.1	0.12						0.03		
Bornyl Acetate	1584	1579	2.89	3.16						0.15	0.11	0.11	0.46		2.22	1.05
Thymol Methyl Ether	1593	1587									0.05	0.11				
Carvacryl Methyl Ether	1597	1598											0.06			
Camphene Hydrate	1603	-														0.05
Terpinen-4-ol	1610	1601	1.12	1.27	0.57	0.39	0.22	0.16	2.10	5.70					0.48	0.54
Terpinen-4-ol Acetate	1626	1640							0.08	0.13						
Cis-Para-Menth-2-en-1-ol	1631	1614			0.3	0.03				0.16					0.02	0.16
Tras-p-Mentha-2,8-dien-1-ol	1634	1639					0.05	0.07								
Myrtenal	1637	1631	0.6	0.65			0.01	0.02			0.04	0.05	0.02	0.03		
Citronellyl Acetate	1661	1657								0.09						
Trans-Pinocarveol	1666	1662	2.15	2.22	0.26	0.06	3.19	3.74	0.04		0.04	0.1	0.03	0.08	0.05	0.05
Cis-Verbenol	1663	1660													0.01	0.01
Cis-Piperitol	1683	-								0.12						
Trans-Verbenol	1688	1681	0.48	0.26									0.01		0.07	0.07
Delta-Terpineol	1678	1679					0.09	0.1							0.09	0.17
Neral	1687	1679					0.04	0.11							0.01	0.02
p-Mentha-1,8-dien-4-ol	1693	1688					0.04	0.01								
α-Terpineol	1699	1695				1.2	0.99	0.92	0.17	0.19	0.1	0.27	0.15	0.13	0.72	1.04
α-Terpinyl Acetate	1707	1695			4.54	4.24			0.61	0.89			0.01			
Borneol	1707	1700	0.61	0.83			0.1	0.12			0.01	0.03	0.01	0.03	2.11	1.91

Verbenone	1723	1721													0.67	0.65
Geranial + Piperitone	1734	1726 + 1730													0.04	0.05
Geranial	1733	1725					0.05	0.03								
Piperitone	1738	1730									0.02	0.02				
Hydroxylinalool I	1745				0.08	0.08										
Trans-Piperitol	1752	1710					0.01									
Carvone	1742	1734	0.06	0.07	0.12										0.01	0.04
Geranyl Acetate	1758	1752			0.45	0.52	0.06	0.02			0.11	0.13				
Trans-Isopiperitenol	1758	1750					0.05	0.06								
Citronellol	1766	1764							0.05							0.01
Myrtenol	1796	1791	0.4	0.46	0.01	0.02	0.05	0.07			0.03	0.04	0.04	0.03	0.02	0.05
Myrtenol + Unknown	1796	1791							0.05							
Trans-Para-Menthadienol	1803	-			0.06	0.05										
Cis-Para-Mentha-1(7),8-dien-2-ol	1803	1811					0.41	0.51								
Trans-2-Trans-4-Decadienal	1826	1808							0.01							
Cis-Sabinol	1822	1801					0.01	0.01								
Geraniol	1847	1840	0.04	0.07		0.61	0.02	0.02			0.01	0.03			0.03	0.03
Trans-Carveol	1839	1836	0.14	0.15	0.03	0.01	0.07	0.11								
Para-Cymen-8-ol	1851	1848	0.04	0.05	0.19		0.02	0.01		0.05			0.01	0.01	0.03	0.03
Cis-Carveol	1847	1855					0.02	0.03								
Cis-p-Mentha-2,8-dien-1-ol	1677	1652					0.03	0.04								
Piperitenone	1929	1918													0.04	0.01
Thymol Isomer	2068	-													0.03	0.02
Thymol	2177	2164					0.02	0.04								0.01

RI_{cal}: retention index obtained in a DB-WAX column; RI_{ref}: retention index based on bibliography; [1]: first sampling period; [2]: second sampling period; C. lad.: *Cistus ladnifer*; E. glob.: *Eucalyptus globulus*; J. com.: *Juniperus communis*; P. pin.: *Pinus pinaster*; P. syl.: *Pinus sylvestris*; R. off.: *Rosmarinus officinalis*.

Table S3. Analysis of the essential oils by GC–MS and GC–FID: sesquiterpene hydrocarbons.

**Component**	**RI_{cal}**	**RI_{ref}**	**C. lad.[1]**	**C. lad.[2]**	**E. glob.[1]**	**E. glob.[2]**	**E. nit.[1]**	**E. nit.[2]**	**J. com.[1]**	**J. com.[2]**	**P. pin.[1]**	**P. pin.[2]**	**P. syl.[1]**	**P. syl.[2]**	**R. off.[1]**	**R. off.[2]**
Isoledene	1476				0.17											
α-Cubebene	1463	1460	0.1	0.1		0.03			0.91	0.15	0.12	0.24	0.09	0.02		
α-Longipinene	1477	1469							0.02		0.18	0.14	0.04	0.01		
α-Ylangene	1491	1484	0.48	0.5					0.01		0.06	0.15		0.04	0.15	0.21
Unknown Sesquiterpene	1491	-		0.1												
Unknown Sesquiterpene	1495	-	0.1													
α-Copaene	1501	1491	0.67	0.79			0.01	0.01	0.46	0.19	0.35	0.6	0.22	0.14	0.07	0.07
Longicyclene	1512	1519									0.05	0.05				
2-Epi-α-Funebrene	1524								0.02							
α-Gurjunene	1543	1530	0.04	0.05	1.68	0.24	0.03	0.03	0.11							
β-Gurjunene	1604	1598			0.47	0.05										
β-Bourbonene	1528	1523							0.04	0.04			0.14	0.14		
Unknown Sesquiterpene	1540	-	0.05													
Unknown Sesquiterpene	1540	-		0.12												
Sativene	1538	-									0.05	0.04				
β-Cubebene	1554	1552							0.13							
Sibirene	1568	-							0.1	0.09						
α-Chamipinene	1572	-							0.14							
1,2-Dihydrocuparene	1584	-							0.08							
α Cedrene + Longifolene	1591	1583 + 1578							0.62							
β-Funebrene	1592	1591							0.73		0.09	0.23	0.01			
Longifolene	1584	1578									2.11	1.70		0.14		
β-Elemene	1604	1591					0.01	0.01	0.93	0.47			0.08	0.08		
α-Barbatene	1598	-							0.05							

β-Copaene	1603	1589									0.06	0.16	0.17	0.15	0.01	0.02
Trans-β Caryophyllene	1617	1598	0.08	0.17			0.1	0.07	3.13	0.39	8.31	12.06	6.87	3.91	3.39	1.93
Aromadendrene isomer	1616	1620			0.36	0.05										
Guaia-6,9-diene	1614	-												0.32	0.03	0.06
Aromadendrene	1618	1620	0.06	0.09	7.20	0.66	0.27	0.37					0.05	0.01		
Isobazzanene	1623	-							0.06							
Thujopsene	1642	1643							12.36	0.06						
γ-Elemene	1643									0.11						
Trans-Muurola-3,5-diene	1644	1250								0.02	0.04	0.09				
Unknown Sesquiterpene	1653	-							0.28							
Unknown Sesquiterpene	1652	-	0.07													
Unknown Sesquiterpene	1652	-		0.08												
Alloaromadendrene	1658	1649	1.28	1.39	1.48	0.23	0.18	0.22								
β-Barbatene	1669	1667							0.07							
Trans-β Farnesene	1673	1671							0.1	0.07	0.1	0.14	0.03	0.02		
Trans-Cadina-1(6),4-diene	1678	-	0.16	0.22					0.16	0.02	0.04	0.1	0.01	0.01		
α-Humulene	1685	1680			0.12	0.04	0.02	0.02	1.9	1.29	1.2	1.79	1.1	0.72	0.53	0.89
Cis-α Bisabolene	1690	1672											0.03			
Maaliene isomer	1693	-							0.08							
β-Acoradiene	1692	1688							0.02							
δ-Elemene	1472	1469											0.77	0.02		
Dehydro Aromadendrane	1703	-	0.22	0.35												
α-Amorphene	1697	1693							0.18		0.49	1.3		0.33	0.16	0.23
Ledene	1705	1704	0.28	0.5	1.34	0.29	0.04	0.05								
Bergamotene isomer	1714	-							0.26							
β-Alaskene	1718	-							0.02							

Germacrene D	1722	1702					0.29	0.02	1.77	1.81	4.05	10.37	6.87	4.33	0.07	0.19
β-Selinene	1732	1725								0.27						
γ-Amorphene	1727	1724									0.15	0.37		0.04		
Unknown sesquiterpene	-	-									0.09	0.23				
Chamigrene Isomer	1728	-							0.19							
γ-Muurolene	1621	-							0.61				0.2	0.08		
β-Bisabolene	1732	1728													0.09	0.13
Valencene	1728	1729							0.33							
α-Muurolene	1742	1740	0.32	0.44			0.05	0.02	0.81		0.3	0.63	0.68	0.92		
α-Selinene	1736	1725	0.06	0.07	0.2	0.13				0.09	0.02	0.05	0.1	0.06		
Bicyclogermacrene + α-Selinene	1747								0.3							
Pseudowiddrene	1754	1745							0.19							
Bicyclogermacrene	1755	1752					0.27	0.34	0.24	0.35			0.28	0.33		
Himachalene Isomer	1759	-							0.4							
α-Chamigrene	1761	1762							0.22							
δ-Cadinene	1774	1772	0.9	1.27	0.12	0.02			2	1.04	0.94	2.09	1.21	0.6	0.02	0.21
γ-Cadinene	1780	1782	0.09	0.16	0.11	0.01	0.02	0.01	1.57	1.14	0.32	0.7	0.51	0.2	0.17	0.05
Ar-Curcumene	1786	1781							0.18						0.04	0.04
Trans-α bisabolene	1779	1775									0.02	0.1				
Selina-3,7(11)-diene	1789	1783							0.02							
Trans-Cadina-1,4-diene	1791	-								0.11	0.05	0.08		0.01		
γ-Cuprenene	1803	-							0.35							
α-Cadinene	1813	1815							0.15		0.05	0.1	0.06	0.03		
δ-Cuprenene	1841	-							0.03							
Trans-Calamenene	1854	1844	0.09	0.12					0.17		0.03	0.02	0.02	0.01	0.01	0.03
Cuparene	1846	1838							0.96							

Germacrene B	1855	1861							0.7	0.96						
α-Calacorene	1924	1921	0.33	0.47					0.04						0.01	0.06

RI_{cal}: retention index obtained in a DB-WAX column; RI_{ref}: retention index based on bibliography; [1]: first sampling period; [2]: second sampling period; C. lad.: *Cistus ladnifer*; E. glob.: *Eucalyptus globulus*; J. com.: *Juniperus communis*; P. pin.: *Pinus pinaster*; P. syl.: *Pinus sylvestris*; R. off.: *Rosmarinus officinalis*.

Table S4. Analysis of the essential oils by GC–MS and GC–FID: oxygenated sesquiterpenes.

Component	RI_{cal}	RI_{ref}	C. lad.[1]	C. lad.[2]	E. glob.[1]	E. glob.[2]	E. nit.[1]	E. nit.[2]	J. com.[1]	J. com.[2]	P. pin.[1]	P. pin.[2]	P. syl.[1]	P. syl.[2]	R. off.[1]	R. off.[2]
Cubebol Isomer I	1895	-								0.03						
Epi-Cubebol	1896	1900	0.11	0.18										0.02		
Cis-Muurol-5-en-4-beta-ol	1906	-							0.04		0.11	0.12				
Palustrol	1944	1938	0.26	0.39		0.04	0.01	0.02								
Trans-Muurol-5-en-4-beta-ol	1950	-	0.07	0.12							0.25	0.2				
Cubebol Isomer II	1959	-							0.03	0.05						
Caryophyllene Oxide (Isomer I)	2007	-					0.01	0.01		0.25	0.26	0.25	0.05			
Caryophyllene Oxide (Isomer II)	2019	-							0.01				0.42			
Caryophyllene Oxide	2002	2014							0.22	0.02				0.12	0.02	0.25
Epi-Globulol	2028	2016			0.62	0.25										
Maaliol	2041	-				0.06	0.01									
Ledol	2052	2057	1.84	2.94		0.07	0.01	0.03								
Trans-Nerolidol	2042	2046					0.02	0.02	0.24	0.15				0.05		
Gleenol	2055	2051							0.01							
Germacrene D-4-ol + Humulene Epoxide	2065	-								0.22						
Germacrene D-4-ol	2065	2050												0.08		
Humulene Epoxide II	2081	2071							0.09				0.14		0.01	0.08
Cubeban-11-ol	2074	-	0.03	0.1		0.08	0.12	0.21								

1,10-Di-Epi-Cubenol	2091	2074							0.02		0.02	0.03	0.02	0.01		
1-Epi-Cubenol	2098	2088	0.07	0.14					0.04	0.02	0.04	0.05	0.03	0.01		
Globulol	2098	2099			2.21	1.41	0.08	0.09								
Guaiol	2107	2096									0.19	0.09				
Viridiflorol	2106	2090	6.40	10.21	0.55	0.37	0.01	0.02								
Unknown Sesquiterpenol	2124	-				0.11										
Rosifoliol	2132	-			0.17	0.22	0.09	0.11								
Unknown Sesquiterpenol	2147	-							0.01							
Spathulenol	2151	-	0.2	0.31		0.04	0.01	0.01	0.08				0.12	0.05		
Alpha-Cedrol	2155	-							0.41							
Widdrol	2182	2178							0.14							
Tau-Cadinol	2193	2180							0.11	0.09			0.06	0.03		
Alpha-Muurolol	2217	2212	0.05	0.15					0.02				0.07	0.02		
T-Muurolol	2204	2187	0.32	0.6			0.01	0.01	0.06	0.03		0.03		0.04		
Alpha-Bisabolol	2229	2228							0.02							
Alpha-Cadinol	2232	2227					0.02	0.01	0.07	0.05	0.04	0.04		0.05		
Beta-Eudesmol	2241	2238	0.12	0.21												
(E,E)-Farnesyl Acetate	2254	2250									0.05	0.05				
(E,E)-Farnesol	2359	2366							0.03	0.03	0.01	0.05				
Unknown Sesquiterpenol	2382	-								0.01						

RI_{cal}: retention index obtained in a DB-WAX column; RI_{ref}: retention index based on bibliography; [1]: first sampling period; [2]: second sampling period; C. lad.: *Cistus ladnifer*; E. glob.: *Eucalyptus globulus*; J. com.: *Juniperus communis*; P. pin.: *Pinus pinaster*; P. syl.: *Pinus sylvestris*; R. off.: *Rosmarinus officinalis*.

Table S5. Analysis of the essential oils by GC–MS and GC–FID: others.

Component	RI_{cal}	RI_{ref}	C. lad.[1]	C. lad.[2]	E. glob.[1]	E. glob.[2]	E. nit.[1]	E. nit.[2]	J. com.[1]	J. com.[2]	P. pin.[1]	P. pin.[2]	P. syl.[1]	P. syl.[2]	R. off.[1]	R. off.[2]
Acetone	791	809					0.01									

1,2,3-Trimethylcyclopentene	733	-	0.02	0.04												
Isovaleraldehyde	903	900			0.05	0.12	0.17	0.75								0.01
1,2,4,4-Tetramethylcyclopentene	920	-	0.23	0.16												
Toluene	1035	1040	0.02	0.02					0.01							
n-Hexanal	1080	1082					0.01	0.01	0.08	0.03				0.01		
Undecane	1120	-									0.02	0.03				
Isoamyl Acetate	1130	1121			0.03	0.03										
Aliphatic ketone	1216	-													0.01	0.01
Trans-2-Hexenal	1220	1216			0.34		0.07	0.03	0.21	0.11						
2-Pentylfuran	1231	1232							0.01						0.02	0.02
Styrene	1251	1261	0.03	0.04												
3-Octanone	1256	1255													3.21	2.42
Isoamyl Isobutyrate	1264	1234								0.11						
Isoamyl Butyrate	1268	1266							0.04							
Hexyl Acetate	1272	1273									0.03	0.03				
2-Methyl Butyl-2-Methyl Butyrate	1276	1276								0.02						
Tridecane	1293	1312									0.25	0.36				
Unknown Alcohol	1314	-	0.07													
Unknown Alcohol	1313	-		0.05												
Cis-3-Hexenyl Acetate	1317	1316					0.65	0.44	0.01	0.01	0.01	0.03	0.07	0.21		
n-Hexanol	1349	1351					0.08	0.02	0.01	0.03						
3-Octanol	1395	1392													0.38	0.17
Cis-3-Hexenol	1380	1380					0.2	0.09	0.01	0.19	0.01	0.02				
n-Nonanal	1395	1391	0.18	0.46			0.01				0.01	0.02		0.03		
Bornyl chloride	1405	-	0.36	0.49												
Trans-2-Hexenol	1407	1400					0.03			0.05						
Isophorone Isomer	1411	-	0.06	0.07												

Hexyl isovalerate	1446	1447									0.01	0.01				
1-Octen-3-ol	1447	1444							0.02	0.05					0.3	0.3
Isoamyl Caproate	1466	1464							0.01	0.02						
Unknown Alcohol	1511	-	0.04													
3-Nonen-2-one	1517	1518	0.31	0.35												
Unknown Alcohol	1510	-		0.03												
Unknown Alcohol	1528	-		0.44												
Unknown Alcohol	1529	-	0.36													
Nopinone	1592	-					0.01	0.01								
Cis-Verbenyl Acetate	1622	-	0.03	0.05												
Beta Cyclocitral	1635	1632	0.04	0.02												
Sabinyl Acetate	1653	1650					0.02	0.03		0.03	0.02	0.01				
Estragole	1671	1671												0.01		
Trans-Pinocarvyl Acetate	1686	1671		0.11												
Cryptone	1687	1675											0.05			
Myrtenyl Acetate	1700	1698	0.54	0.61					0.02	0.06						
Carvyl Acetate	1741	-	0.13	0.14												
Geranyl Propanoate	1820	1816	0.05	0.08												
Phenethyl Acetate	1821	1825					0.01	0.01								
Trans-Anethole	1835	1826														0.02
Phenethyl Alcohol	1916	1904					0.01	0.01								
Geranyl Isovalerate	1919	1904									0.02	0.02				
n-Dodecanol	1964	1959												0.04		
Phenethyl-2-Methyl Butyrate	1980	1968									0.12	0.18		0.01		
Phenethyl Isovalerate	1996	-				0.1	0.01	0.2			0.22	0.3				
Methyl Eugenol	2014	2006					0.01	0.01			0.08	0.11		0.05	0.25	0.01
Eugenol	2159	2163														0.02

Mint sulfide	2187	-								0.15			
Cembrene Isomer	2199	2207											0.06
Ambrox	2200	-	0.04	0.19									
Sclareoloxide	2270	-	0.26										
Unknown Epoxide	2482	-						0.09					
Rimuene	2351	-								1.63	0.35		
Abieta-8,12-diene	2368	-								1.33	0.35		
Abietadiene	2437	2450								4.34	0.85		
Abietatriene	2526	2524								0.87	0.06		
Unknown alcohol		-											0.3
Neoabietadiene	2598	-								0.76			

RI_{cal}: retention index obtained in a DB-WAX column; RI_{ref}: retention index based on bibliography; [1]: first sampling period; [2]: second sampling period; C. lad.: *Cistus ladnifer*; E. glob.: *Eucalyptus globulus*; J. com.: *Juniperus communis*; P. pin.: *Pinus pinaster*; P. syl.: *Pinus sylvestris*; R. off.: *Rosmarinus officinalis*.
